# Spherical trihedral metallo-borospherenes

**DOI:** 10.1038/s41467-020-16532-x

**Published:** 2020-06-02

**Authors:** Teng-Teng Chen, Wan-Lu Li, Wei-Jia Chen, Xiao-Hu Yu, Xin-Ran Dong, Jun Li, Lai-Sheng Wang

**Affiliations:** 1 Department of Chemistry, Brown University, Providence, RI 02912 USA; 2 Department of Chemistry and Key Laboratory of Organic Optoelectronics & Molecular Engineering of Ministry of Education, Tsinghua University, 100084 Beijing, China; 3 Institute of Theoretical and Computational Chemistry, Shaanxi Key Laboratory of Catalysis, School of Chemical & Environment Sciences, Shaanxi University of Technology, 723000 Hanzhong, China; 4 Department of Chemistry, Southern University of Science and Technology, 518055 Shenzhen, China

**Keywords:** Chemical bonding, Chemical physics

## Abstract

The discovery of borospherenes unveiled the capacity of boron to form fullerene-like cage structures. While fullerenes are known to entrap metal atoms to form endohedral metallofullerenes, few metal atoms have been observed to be part of the fullerene cages. Here we report the observation of a class of remarkable metallo-borospherenes, where metal atoms are integral parts of the cage surface. We have produced La_3_B_18_^–^ and Tb_3_B_18_^–^ and probed their structures and bonding using photoelectron spectroscopy and theoretical calculations. Global minimum searches revealed that the most stable structures of Ln_3_B_18_^–^ are hollow cages with *D*_3*h*_ symmetry. The B_18_-framework in the Ln_3_B_18_^–^ cages can be viewed as consisting of two triangular B_6_ motifs connected by three B_2_ units, forming three shared B_10_ rings which are coordinated to the three Ln atoms on the cage surface. These metallo-borospherenes represent a new class of unusual geometry that has not been observed in chemistry heretofore.

## Abstract (web summary)

Borospherenes are the boron-based analogs of fullerene cages. Here, the authors report a class of Ln_3_B_18_^–^ metallo-borospherenes with unusual spherical trihedron geometry, in which the lanthanide atoms surprisingly form a part of the cage surface.

## Introduction

The electron deficiency of boron often leads to electron delocalization and the violation of the octet rule in boron compounds and three-dimensional (3D) cage units in different bulk boron allotropes^1–3^. Because of the strong boron–boron bonding, there were speculations about the formation of boron nanotubes composed of a triangular boron lattice^4,5^, after the discovery of carbon nanotubes. The triangular boron lattice can be viewed as a graphene-like sheet with the filling of a boron atom in each B_6_ hexagon. Further theoretical calculations revealed, however, that triangular lattices with hexagonal vacancies were more stable and more suitable to construct boron nanotubes^6,7^. In the meantime, combined spectroscopic and theoretical studies have shown that size-selected boron clusters all have 2D structures with delocalized multi-center bonding within the cluster plane^8–11^. The discovery of the hexagonal 2D B_36_ clusters provided the first experimental evidence of the viability of atom-thin boron nanostructures with hexagonal vacancies, named as borophene akin to graphene^12^. Borophenes have been recently synthesized using atomic vapor deposition on Ag(111) substrates^13,14^, forming a new class of synthetic 2D nanomaterials^15^. The analogy between nanostructures made of boron and carbon has been further extended when the B_40_ and B_39_^–^ clusters were found to have global minimum cage structures^16,17^, i.e. borospherenes analogous to the fullerenes. Fullerenes are known to form endohedral metallofullerenes for alkali, alkali earth, lanthanide, and actinide elements^18,19^, albeit not for transitions metals. Heterofullerenes in which one carbon atom is substituted by a transition metal atom have been observed in the gas phase, but the metal substitution induces large local structural distortions and such heterofullerenes have not been synthesized in the bulk^20–22^. The first cage cluster made of multiple metal atoms and carbons was proposed to be Ti_8_C_12_^+^ (metallocarbohedrene)^23^. However, subsequent theoretical calculations showed that the metallocarbohedrene is not stable and the global minimum of Ti_8_C_12_^+^ consisted of a tetrahedral, close-packed Ti_8_ clusters coordinated by six C_2_ units on the cluster surface^24^. In the present article, we report the first observation of a class of metallo-borospherenes, hollow cage clusters consisting of three lanthanide (Ln) atoms and 18 boron atoms (Ln_3_B_18_^–^).

Transition-metal-doped boron clusters were first found to form aromatic borometallic molecular wheels, M©B_*n*_^−^ (*n* = 8–10)^25,26^, as well as metal-centered nanotubular structures^27^. More interestingly, it has been shown that transition metals can be an integral part of larger 2D boron clusters^28^, leading to the possibility of metallo-borophenes^29^. Lanthanide-doped boron clusters, however, have been found recently to form very different structures, due to both charge transfer interactions and strong (*d–p*)*π* bonding^30^. For example, lanthanide-doped boron clusters do not form similar borometallic molecular wheels as the transition metals. Instead, they form inverse-sandwich-type structures for Ln_2_B_*n*_^–^ clusters (*n* = 7–9)^31,32^. The most recent study indicates that the inverse-sandwich structure may extend to form lanthanide-boron nanowires^33^.

Here we report a joint photoelectron spectroscopy (PES) and quantum chemistry study of two tri-lanthanide-doped B_18_ clusters (La_3_B_18_^–^ and Tb_3_B_18_^–^), which are found to possess unprecedented *D*_3*h*_ cage structures with the Ln atoms being integral parts of the cage surface. These *D*_3*h*_ metallo-borospherenes belong to an unusual class of geometry known as spherical trihedron. The B_18_ framework consists of two B_6_ triangles connected by three B_2_ units, forming three shared B_10_ rings. The high stability of the spherical trihedral structures is derived from the strong interactions between the Ln atoms and the B_10_ rings via charge transfer interactions and *d*–*p* covalent bonding. Theoretical calculations show that the entire series of lanthanide elements (Ln = La–Lu) can form spherical trihedral Ln_3_B_18_^–^ metallo-borospherenes with tunable magnetic properties, making them a fascinating series of building blocks for new types of magnetic materials.

## Results and discussion

### Photoelectron spectroscopy

**Fig. 1:**
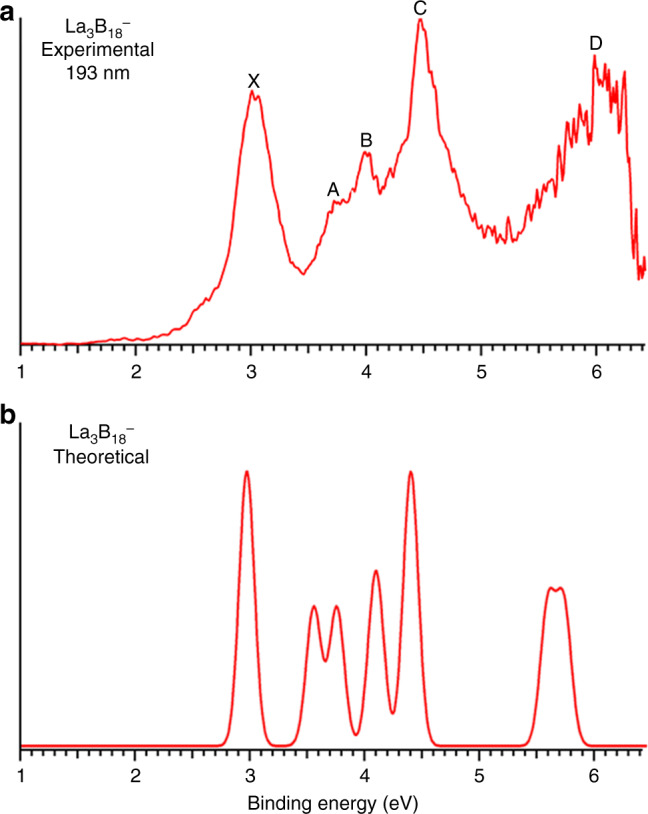
Photoelectron spectrum of La_3_B_18_^–^. **a** At 193 nm. **b** The simulated spectrum.

**Fig. 2:**
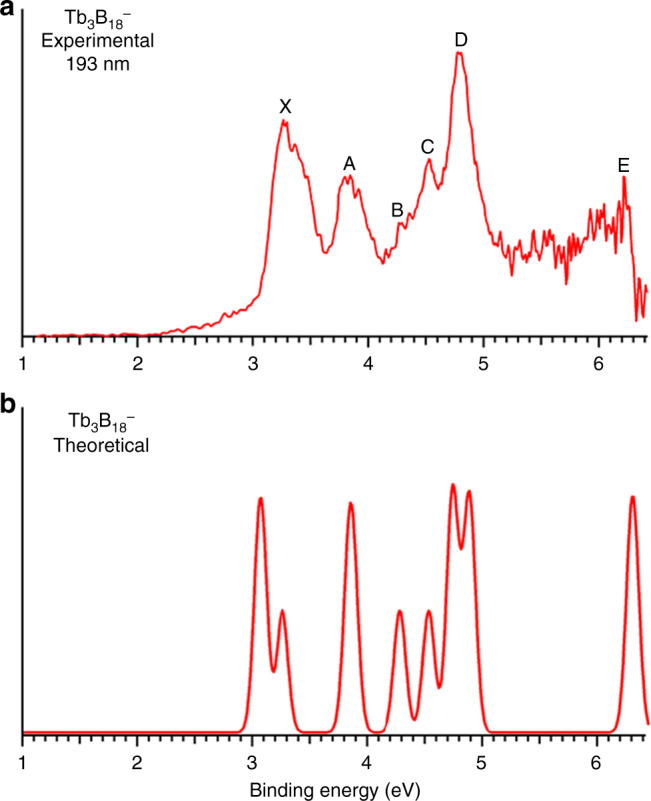
Photoelectron spectrum of Tb_3_B_18_^–^. **a** At 193 nm. **b** The simulated spectrum.

The PE spectrum at 193 nm was first measured for the La_3_B_18_^–^ cluster (Fig. 1a), which was found to exhibit a relatively simple pattern compared with that of the recently reported La_3_B_14_^–^ cluster^33^. This observation suggested that La_3_B_18_^–^ should possess a highly symmetric structure. Subsequently, we also obtained the spectrum of a late-Ln cluster Tb_3_B_18_^–^ (Fig. 2a) and observed a spectral pattern, exhibiting some similarities to that of La_3_B_18_^–^ and suggesting that these two Ln-doped boron clusters should have similar structures and chemical bonding. The well-resolved PES features of the Ln_3_B_18_^–^ clusters serve as electronic fingerprints to allow analyses of their structures and chemical bonding by comparing with theoretical calculations, as shown in Figs. 1b, 2b, and Supplementary Tables S1 and S2 for Ln = La and Tb, respectively.

The spectrum of La_3_B_18_^–^ displayed five well-resolved bands labeled as X, A, B, C, and D (Fig. 1a). The X band yielded the first vertical detachment energy (VDE) of 2.97 eV for La_3_B_18_^–^. The adiabatic detachment energy (ADE) for band X was evaluated from its onset to be 2.80 eV, which also represents the electron affinity (EA) of neutral La_3_B_18_. The higher binding energy bands (A, B, C, and D) correspond to detachment transitions to the excited states of neutral La_3_B_18_. The A band at 3.64 eV was broad and not well resolved at 193 nm, but it was slightly better resolved in the 266 nm spectrum (Supplementary Fig. 1). This broad spectral feature could be due to geometry changes upon electron detachment or overlapping detachment transitions. Band B at 4.01 eV is sharper compared with band A (Fig. 1a). An intense and sharp band C at 4.43 eV was clearly resolved in the 193 nm spectrum. Following a large energy gap, a broad band (D) was observed above ~5.5 eV. Due to the poor signal-to-noise ratio, band D was tentatively assigned for the sake of discussion.

The PE spectrum of Tb_3_B_18_^–^ showed five well-resolved peaks assigned as X, A, B, C, D (Fig. 2a). The X band gave rise to a VDE of 3.26 eV for Tb_3_B_18_^–^ and an ADE of 3.13 eV, which is also the EA of neutral Tb_3_B_18_. Band A was observed at a VDE of 3.84 eV, followed by three closely-lying bands (B, C, D). Band B at 4.28 eV and band C at 4.52 eV were relatively weak and closely spaced, whereas band D at 4.77 eV was much more intense. Beyond ~5 eV, the signal-to-noise ratio was poor and no obvious spectral bands were observed. Band E close to the threshold at a VDE of ~6.2 eV was tentatively labeled. The overall spectral pattern for Tb_3_B_18_^–^ exhibits some similarity to that of La_3_B_18_^–^. In particular, the strong X and D bands in Tb_3_B_18_^–^ are similar to the strong X and C bands in La_3_B_18_^–^. There is a large energy gap on the high binding energy side in both spectra. Similar spectral patterns could be an indication of similar structures, as have been observed for a series of dilanthanide clusters (Ln_2_B_8_^–^)^31^.

### Global minimum structural searches

**Fig. 3:**
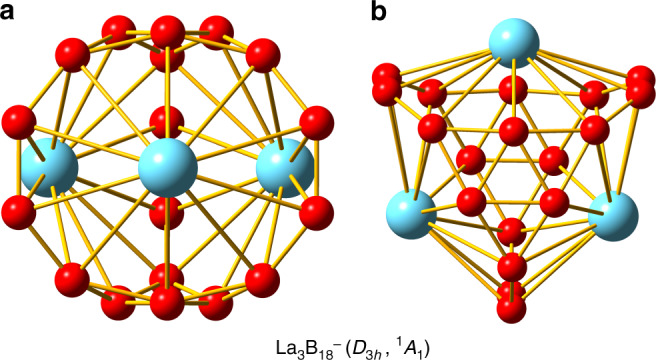
The global minimum structure of La_3_B_18_^–^ (*D*_3*h*_, ^1^*A*_1_) at the PBE0/TZP level. **a** The *C*_3_ axis is along the page vertically. **b** The *C*_3_ axis is perpendicular to the page.

The low-lying isomers within 55 kcal mol^−1^ of the global minimum at the levels of PBE/TZP and PBE0/TZP are presented in Supplementary Fig. 2. The global minimum of La_3_B_18_^–^ is a hollow cage with a closed-shell ground state (^1^*A*_1_) and *D*_3*h*_ symmetry. This is a hetero-metallo-borospherene, in which the three La atoms are integral parts of the cage surface, as shown in Fig. 3. All the other low-lying isomers are low-symmetry 3D structures, many of which are distorted cages. The highly symmetric global minimum *D*_3*h*_ metallo-borospherene exhibits overwhelming stability relative to the other low-lying isomers: it is more stable than the nearest isomer with *C*_*s*_ symmetry by ~19 kcal mol^−1^ at the PBE/TZP and PBE0/TZP levels of theory. The B_18_ framework in the La_3_B_18_^–^ cage can be viewed as consisting of two B_6_ triangles linked together at their three corners by three B_2_ units, creating three shared B_10_ rings along the *C*_3_ axis. The three La atoms are coordinated by the three B_10_ rings, giving rise to the closed cage structure. The La_3_B_18_^–^ metallo-borospherene has an oblate shape with a diameter of 4.62 Å along the *C*_3_ axis (between the two B_6_ triangles) and 5.09 Å encompassed by the three equatorial La atoms. The relevant bond lengths of the La_3_B_18_^–^ metallo-borospherene are shown in Supplementary Fig. 3a.

The hollow cage structure of La_3_B_18_^–^ was totally unexpected and the geometry is highly unusual. To further examine its stability and robustness, we performed ab initio molecular dynamics (AIMD) simulations at different temperatures, 300, 500, 700, and 1000 K (see Supplementary Fig. 4). We found that even at 1000 K the La_3_B_18_^–^ metallo-borospherene is dynamically stable for the 13 ps duration of the simulations. At 1000 K, the structure displayed a root-mean-square-deviation of 0.199 Å and a maximum bond length deviation of 0.260 Å during the simulations.

The similarity in their PE spectra suggested that the global minima of Tb_3_B_18_^–^ and La_3_B_18_^–^ should be similar. Because of the localized and nonbonding nature of the 4*f* orbitals in Tb, we optimized the *D*_3*h*_ structure for Tb_3_B_18_^–^ using the 4*f*-in-core pseudopotential^34^. The structural parameters of the Tb_3_B_18_^–^ metallo-borospherene are similar to those for La_3_B_18_^–^ (Supplementary Fig. 3), except that the Tb–B and B–B bond lengths are all slightly shorter due to the smaller atomic radius of Tb as a result of the lanthanide contraction. Because of the use of the 4*f*-in-core pseudopotential, the spin state of the Tb_3_B_18_^–^ metallo-borospherene was not determined from the geometry optimization. We performed broken symmetry calculations and compared the relative energies between the ferromagnetic and antiferromagnetic couplings of the 4*f* electrons, as shown in Supplementary Table 3 for Tb_3_B_18_^–^, as well as for Pr_3_B_18_^–^. The relative energies due to the inter-atomic spin couplings of the unpaired 4*f* electrons are relatively small, although the high spin ferromagnetic coupling seems to give the lowest energy in both cases. Hence, the spin multiplicity of the Tb_3_B_18_^–^ metallo-borospherene should be 19 (with 18 unpaired 4*f* electrons).

### Comparison between the experimental and theoretical results

**Fig. 4:**
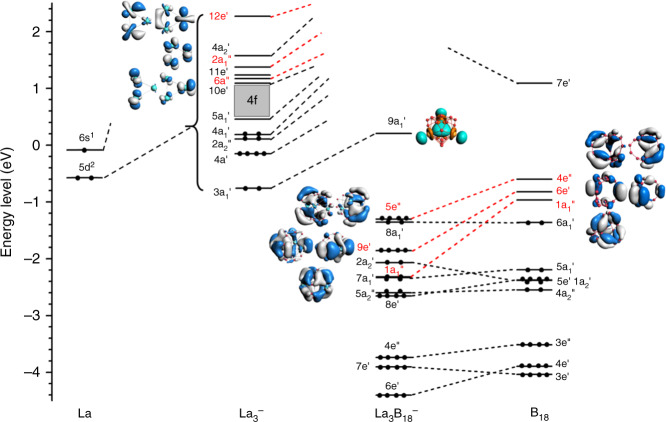
The Kahn–Sham molecular orbital correlation diagram for La_3_B_18_^–^ (*D*_3*h*_, ^1^*A*_1_). It shows the interactions between the 5*d* orbitals of the three La atoms and the group orbitals of the B_18_ moiety.

To validate the *D*_3*h*_ cage structure for La_3_B_18_^–^ and Tb_3_B_18_^–^, we calculated their ADEs and VDEs using the ΔSCF–TDDFT formalism. Figures 1b, 2b present the simulated spectra for the *D*_3*h*_ global minimum structures, in comparison with the experimental data. The computed ADE/VDE_1_ at the CCSD(T) level are 2.828/2.972 eV for La_3_B_18_^–^ (Supplementary Table 4), in excellent agreement with the experimental data of 2.80/2.97 eV. As shown in Fig. 4, the valence MOs of La_3_B_18_^–^ are mainly of La-B *d–p* and B *sp* characters. Because La_3_B_18_^–^ has a closed-shell configuration, single-electron removal from each molecular orbital (MO) yields one detachment channel, as shown in Supplementary Table 1. The computed VDEs for detachment from the 5*e*′′ HOMO (2.972 eV) and 8*a*_1_′ HOMO-1 (2.987 eV) are very close to each other, in excellent agreement with the experimental VDE of the X band (2.97 eV). In fact, each of the observed PES band corresponds to two detachment channels, as given in Supplementary Table 1, where the electron configurations and final state symmetries are also presented. The simulated spectral patterns and the experimental spectra are in excellent agreement, providing considerable credence for the *D*_3*h*_ cage global minimum for La_3_B_18_^–^. We have also simulated the PE spectra for the next nine higher-lying isomers of La_3_B_18_^–^, as shown in Supplementary Fig. 5. None of these spectra fits the experimental spectrum, giving additional support for the *D*_3*h*_ global minimum structure.

The computed ADE/VDE for Tb_3_B_18_^–^ are 2.901/3.017 eV at the CCSD(T) level (Supplementary Table 4), slightly underestimated relative to the experimental data of 3.13/3.26 eV probably due to the use of the 4*f*-in-core approximation as well as the incomplete account of electron correlations. Nevertheless, the theoretical results by not considering the 4*f* electrons and detachment channels are still in very good agreement with the experimental data, as can be seen in Fig. 2 and Supplementary Table 2. These results are consistent with our previous observations that the detachment cross sections of the 4*f* electrons are much weaker and the PE spectra of Ln–B binary clusters are dominated by the Ln–B *d–p* and B *sp* detachment channels^31,32^.

### Stabilities of the first metallo-borospherenes

The observation of the La_3_B_18_^–^ and Tb_3_B_18_^–^ cage clusters, in which the three Ln atoms are integral parts of the cage surface, is unprecedented. The two B_6_ triangles in the *D*_3*h*_ structure are reminiscent of the B_40_ borospherene^16^, which consists of eight fused B_6_ triangles on a spherical surface. Hence, the Ln_3_B_18_^–^ cage clusters can be viewed as a new class of metallo-borospherenes. Networked metallo-fullerenes usually involve a single transition-metal atom substituting one carbon atom on the fullerene surface^20–22^. The incorporation of multiple metal atoms on the borospherene surface is due to the flexibility of the 2D boron network, which is a direct result of the electron deficiency of boron. It is interesting to note that the crystal structure of a Ni–Zn boride (Ni_21_Zn_2_B_24_) was shown to contain characteristic cages of B_20_ units, with an octahedral Ni_6_ cluster nested inside^35^. Our observation of the Ln_3_B_18_^–^ metallo-borospherenes represents the first isolated molecules of Ln–B cages in the gas phase.

**Fig. 5:**
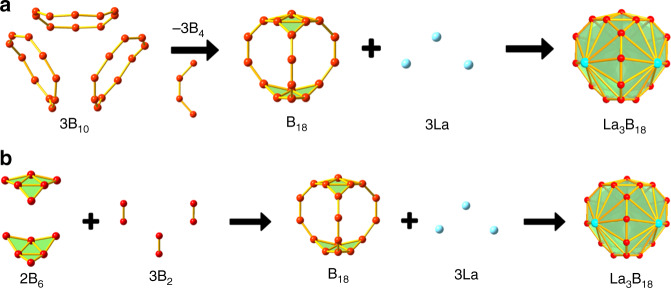
Schematic pathways for the formation of the *D*_3*h*_ Ln_3_B_18_^–^ metallo-borospherenes. Two pathways for the construction of the B_18_ framework and its bonding with the three Ln atoms are depicted. **a** The fused B_10_ ring pathway. **b** The B_2_-linked triangular B_6_ pathway.

To understand the stability of these remarkable cage structures, we carried out fragment MO analyses by first considering the construction of the B_18_ framework in two different pathways and then its bonding with the three La atoms, as schematically shown in Fig. 5. Figure 5a shows one possible path to construct the B_18_ framework by the fusion of three B_10_ rings. Four of the boron atoms in each B_10_ ring are shared with the other B_10_ rings, which each coordinate to a La atom to form three shared La©B_10_ units. This hypothetical formation pathway of La_3_B_18_^–^ can be expressed by the following steps:

1 $$3{\mathrm{B}}_{10} \to {\mathrm{B}}_{18} + {\mathrm{3B}}_4\;\;\;\;\Delta E_1 = 120.1\;{\mathrm{kcal}}\;{\mathrm{mol}}^{ - 1}$$

2 $${\mathrm{B}}_{18} + {\mathrm{3La}} \to {\mathrm{La}}_{\mathrm{3}}{\mathrm{B}}_{18}\;\;\;\;\Delta E_2 = - 655.8\;{\mathrm{kcal}}\;{\mathrm{mol}}^{ - 1}$$

3 $${\mathrm{La}}_{\mathrm{3}}{\mathrm{B}}_{{\mathrm{18}}} + {\mathrm{e}}^- \to {\mathrm{La}}_{\mathrm{3}}{\mathrm{B}}_{18}^-\;\;\;\;\Delta E_3 = - 68.8\;{\mathrm{kcal}}\;{\mathrm{mol}}^{ - 1}$$

4 $$3{\mathrm{B}}_{10} + {\mathrm{3La}} + {\mathrm{e}}^- \to 3{\mathrm{B}}_4 + {\mathrm{La}}_{\mathrm{3}}{\mathrm{B}}_{18}^-\;\;\;\Delta E_{\mathrm{A}} = - 604.5\;{\mathrm{kcal}}\;{\mathrm{mol}}^{ - 1}$$

The energetics were calculated from single-point energy differences of the reactants and products, using the geometries directly taken from the optimized La_3_B_18_^–^ cage at the PBE/TZP level of theory. The interactions between the B_18_ framework and the three La atoms are extremely strong (steps 2 and 3), which underlies the stability of the La_3_B_18_^–^ cage. It should be noted that in platonic solids four surfaces are the minimum number to form a 3D object, i.e., the tetrahedron. However, Fig. 5a shows that three La©B_10_ surfaces are fused together to form the *D*_3*h*_ La_3_B_18_^–^ cage. This is because the La©B_10_ surface is curved. It turns out that the Ln_3_B_18_^–^ metallo-borospherenes belong to a class of geometry mathematically known as *n*-gonal hosohedron, which is basically a tessellation of lunes on a spherical surface, such that each lune shares the same two vertices. Thus, the *D*_3*h*_ La_3_B_18_^–^ cage is a trigonal hosohedron, also known as spherical trihedron, where the two vertices consist of the triangular B_6_ units. To the best of our knowledge, such a geometry has not been observed in any cluster or molecular systems heretofore.

Figure 5b shows another pathway to construct the La_3_B_18_^–^ cage, in which the B_18_ framework is formed by two triangular B_6_ motifs linked by three B_2_ bridges, such that three B_10_ rings are created. This hypothetical pathway can be represented by the following steps:

5 $$2{\mathrm{B}}_{\mathrm{6}} + 3{\mathrm{B}}_2 \to {\mathrm{B}}_{18}\;\;\;\;\Delta E_5 = - 707.9\;{\mathrm{kcal}}\;{\mathrm{mol}}^{ - 1}$$

6 $${\mathrm{B}}_{18} + {\mathrm{3La}} \to {\mathrm{La}}_{\mathrm{3}}{\mathrm{B}}_{18}\;\;\;\;\Delta E_6 = - 655.8\;{\mathrm{kcal}}\;{\mathrm{mol}}^{ - 1}$$

7 $${\mathrm{La}}_{\mathrm{3}}{\mathrm{B}}_{{\mathrm{18}}} + {\mathrm{e}}^- \to {\mathrm{La}}_{\mathrm{3}}{\mathrm{B}}_{{\mathrm{18}}}^-\;\;\;\;\Delta E_7 = - 68.8\;{\mathrm{kcal}}\;{\mathrm{mol}}^{ - 1}$$

8 $$2{\mathrm{B}}_6 + {\mathrm{3B}}_2 + {\mathrm{3La}} + e^- \to {\mathrm{La}}_{\mathrm{3}}{\mathrm{B}}_{18}^-\;\;\;\;\Delta E_B = - 1432.5\;{\mathrm{kcal}}\;{\mathrm{mol}}^{ - 1}$$

The interactions between the B_18_ framework and the three La atoms to form the La_3_B_18_^–^ cage are represented by steps 2/3 or 6/7 with an estimated binding energy of 724.6 kcal mol^-1^, i.e., 241.5 kcal mol^−1^ for the binding energy between each La atom and the B_10_ ring. This huge La-B_10_ binding energy underlies the extraordinary stability of the La_3_B_18_^–^ metallo-borospherene. Compared with the pathway in Fig. 5a, the pathway in Fig. 5b is more favorable energetically since each step is exothermic. We should emphasize, though, that these exercises provide different views of the unprecedented hollow cage structures. The two pathways to construct the La_3_B_18_^–^ cage depicted in Fig. 5 certainly do not represent the mechanisms about how it is formed.

### The nature of the bonding between B_18_ and the La atoms

Since the global minimum of B_18_ is a planar structure^36^, the stabilization of the 3D B_18_ framework is entirely due to its strong bonding with the three La atoms, as discussed above. We have analyzed the nature of the La–B_10_ bonding in the La_3_B_18_^–^ metallo-borospherene using several different methods. The MO energy-level diagram and the relevant MOs of La_3_B_18_^–^ derived from the La_3_^–^ and B_18_ moieties are shown in Fig. 4. The 5*e*′′, 9*e*′, and 1*a*_1_′′ MOs of La_3_B_18_^–^ (red-colored) represent the bonding orbitals between the three La atoms and the B_18_ moiety, mainly corresponding to the interactions between the irreducible representations, 4*e*′′, 6*e*′, and 1*a*_1_′′ on the B_18_ moiety and 6*e*′′, 12*e*′, and 2*a*_1_′′ on the La_3_^–^ moiety (the red highlighted MOs). Supplementary Table S5 gives the compositions of the 5*e*′′, 9*e*′, and 1*a*_1_′′ MOs, which are dominated by contributions from the B_18_ moiety. Hence, there is a strong charge transfer from La to B_18_, resulting in a closed-shell La_3_B_18_^–^ with a large HOMO-LUMO gap of 1.51 eV computed at the PBE/TZP level. The La atoms are in their favorite +III oxidation state in La_3_B_18_^–^, which can be viewed approximately as (La^3+^)_3_[B_18_^10–^]. As shown in Fig. 4, the 6*e*′′, 12*e*′, and 2*a*_1_′′ irreducible representations on the La_3_^–^ moiety are of La 5*d* characters, while the 4*e*′′, 6*e*′, and 1*a*_1_′′ irreducible representations on the B_18_ framework are of B 2*p* characters. Hence, the 5*e*′′, 9*e*′, and 1*a*_1_′′ MOs also represent significant La 5*d* and B_18_ 2*p* covalent bonding. It is the strong covalent and ionic bonding between the La atoms and the B_10_ rings that gives rise to the extraordinary stability of the La_3_B_18_^–^ cage structure. These bonding characteristics are found in all lanthanide boride compounds due to the low electronegativity of the lanthanide elements and their diffuse 5*d* orbitals^37^.

The La–B_10_ interactions can be further characterized using the EDA-NOCV method with B_18_ (…6*a*_1_′^2^1*a*_1_′′^0^6*e*′^0^4*e*′′^0^) and La_3_^–^ (…6*e*′′^4^2*a*_1_′′^2^12*e*′^4^) fragments, a powerful energy decomposition tool to give insight into chemical bonding^38^. We analyzed the B_18_…La_3_^–^ interaction by the decomposition of the orbital terms into pairwise contributions, as shown in Supplementary Fig. 6. There are three major terms Δ*E*_1_, Δ*E*_2_, and Δ*E*_3_ associated with the deformation densities Δ*ρ*_1_, Δ*ρ*_3_, and Δ*ρ*_3_, respectively. The remaining terms contribute <10% to the total orbital interactions. The color code of the deformation densities indicates the direction of the charge flow from red → blue. It is interesting to see that the 1*a*_1_′′ orbital of La_3_B_18_^–^, which is analogous to the (*d–p*)*δ* bonding MO in the Ln_2_B_8_^–^ inverse sandwich complexes^31,32^, contributes significantly (25.7% from the EDA-NOCV analyses, Supplementary Fig. 6) to the stability of the orbital interaction. The other two stronger Δ*ρ*_2_ (34.8%) and Δ*ρ*_3_ (28.9%) deformation densities correspond to the degenerate 9*e*′ and 5*e*′′ MOs, respectively. The direction of the charge flow is from the La_3_^–^ to the B_18_ moiety, consistent with the fragment MO analyses discussed above (Fig. 4).

**Fig. 6:**
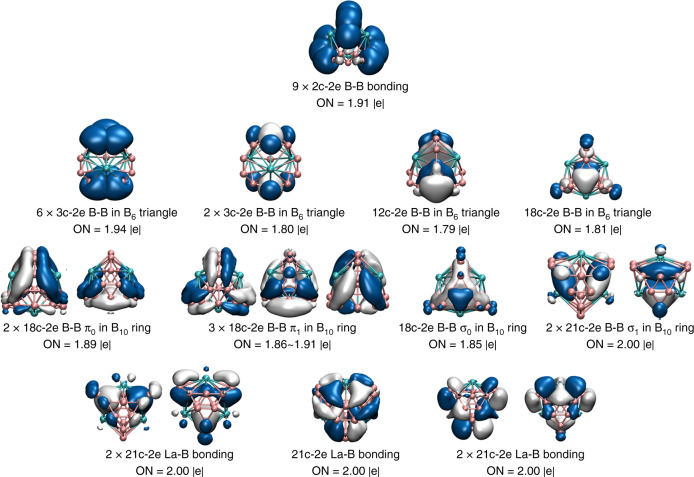
Chemical bonding analyses of La_3_B_18_^–^ (*D*_3*h*_^1^*A*_1_). The analyses were done using the AdNDP method^29^. ON stands for occupation number.

We further analyzed the chemical bonding in the La_3_B_18_^–^ metallo-borospherene using the adaptive natural density partitioning (AdNDP) approach^39^, as shown in Fig. 6. The first row displays nine localized two-center two-electron (2c–2e) *σ* bonds formed within the three B_2_ units and between the B_2_ units and the three apexes of the two B_6_ triangles. The second row reveals the delocalized bonds in the B_6_ triangles, with four three-center two-electron (3c–2e) *σ* bonds within each B_6_ unit. The multi-center 12c–2e and 18c–2e delocalized bonds can be viewed as *π* bonds within the B_6_ units. The third row represents totally delocalized σ and π bonds within the B_18_ framework. The last row shows five totally delocalized 21c–2e bonds between the La atoms and the B_18_ framework, corresponding to the 5*e*′′, 9*e*′, and 1*a*_1_′′ MOs in Fig. 4. We also found that the La_3_B_18_^–^ metallo-borospherene possesses both 3D aromaticity with calculated nucleus-independent chemical shifts (NICS)^40^ of −47.87 ppm at the cage center, and planar aromaticity on each B_6_ triangles with NICS(0) of −31.44 ppm and NICS(1) of −2.16 above the plane center, as shown in Supplementary Table 6, where the aromaticity in the metallo-borospherene is compared with that of the recently synthesized cubic [Zn^I^]_8_ compound^41^.

We also performed bond-order index analyses for the B–B and La–B interactions, as presented in Supplementary Table 7. The B_2_ bridges have shorter bond lengths and higher bond orders than those of the B_6_ triangles. In terms of the La–B interactions, the distances and bond order indices are similar to those in the lanthanide–boron complexes reported previously^30–33^.

### A new class of spherical trihedron metallo-borospherenes

The discoveries of the La_3_B_18_^–^ and Tb_3_B_18_^–^ metallo-borospherenes suggest that other lanthanide elements could also form similar structures because of the similarity in the chemical properties of the whole series of lanthanides. We have calculated the *D*_3*h*_ cage structures for all the lanthanide elements, Ln_3_B_18_^–^ (Ln = Ce–Lu). The coordinates obtained at the PBE0 level are given as Supplementary Data 1, whereas those of La_3_B_18_^–^ and La_3_B_18_ are provided in Supplementary Table 8. All these structures are indeed minima on their potential energy surfaces. Hence, we conclude that there indeed exist a whole class of Ln_3_B_18_^–^ metallo-borospherenes. While borospherenes have not been observed beyond the B_40_ cluster^42^, the unique bonding characteristics between lanthanide and boron suggest that other lanthanide metallo-borospherenes with different sizes and Ln_*x*_B_*y*_^–^ stoichiometries may exist. Recent studies of transition-metal borides showed that the metal–boron interactions have major influences on their magnetic properties^43,44^. Hence, the understanding of Ln–B interactions in the metallo-borospherene systems may provide insights for the design of lanthanide borides with tunable magnetic or catalytic properties.

In conclusion, we report the observation of the first tri-lanthanide-doped boron cage clusters (metallo-borospherenes), in which the metal atoms are integral parts of the cage surface. Photoelectron spectra of two representative systems, Ln_3_B_18_^–^ (Ln = La, Tb), show similar and relatively simple spectral patterns, suggesting that they have similar highly symmetric structures. Theoretical calculations reveal that the Ln_3_B_18_^–^ anions have cage-like structures with *D*_3*h*_ symmetry: two planar B_6_ triangular units linked by three B_2_ bridges to form the B_18_ framework consisting of three shared B_10_ rings coordinated to the three Ln atoms. Strong ionic and covalent chemical bonding is found between the Ln atoms and the B_18_ framework. The extraordinary stabilities of the metallo-borospherenes are understood by various theoretical analyses. La_3_B_18_^–^ is found to have a closed-shell electron configuration with a large HOMO-LUMO gap and possesses 3D aromaticity. The Ln_3_B_18_^–^ cage complexes are expected to exist for all lanthanide elements, suggesting the possibility that there may exist a large class of lanthanide metallo-borospherenes with different Ln/B stoichiometries and tunable properties.

## Methods

### Experimental details

The experiments were carried out using a magnetic-bottle PES apparatus equipped with a laser vaporization supersonic cluster source, details of which have been published elsewhere^11^. The La_3_B_18_^–^ and Tb_3_B_18_^–^ clusters were produced by laser vaporization of a La/^11^B or Tb/^11^B mixed target, respectively. The laser-induced plasma was cooled by a He carrier gas seeded with 5% Ar, initiating nucleation between the boron and lanthanide atoms. The nascent clusters were entrained in the carrier gas and underwent a supersonic expansion. Negatively-charged clusters were extracted from the collimated cluster beam and analyzed by a time-of-flight mass spectrometer. Both pure (B_*n*_^–^) and mixed (Ln_*x*_B_*y*_^–^) clusters were produced from the cluster source. The La_3_B_18_^–^ and Tb_3_B_18_^–^ clusters of current interest were mass-selected and photodetached by the 193 nm (6.424 eV) radiation from an ArF excimer laser or the fourth harmonics from a Nd:YAG laser (266 nm, 4.661 eV). Photoelectrons were collected and analyzed in a 3.5-m-long electron flight tube at nearly 100% efficiency. The photoelectron spectra were calibrated by the known transitions of Au^–^ and Bi^–^. The resolution of the PES apparatus (ΔKE/KE) was around 2.5%, that is, about 25 meV for photoelectrons with 1 eV kinetic energy (KE).

### Computational methods

Unbiased global-minimum structural searches for the La_3_B_18_^–^ cluster were performed using the TGMin 2.0 code^45^. The global minimum structure of Tb_3_B_18_^–^ was not searched separately. More than 2000 structures were evaluated for La_3_B_18_^–^ using the constrained Basin–Hopping algorithm at the PBE/DZP^46,47^ level from the ADF 2017 software^48^. A *D*_3*h*_ cage structure was found to be the global minimum, which was significantly lower in energy in comparison to the next lowest-lying isomer (Supplementary Fig. 2). To confirm the stability of the global minimum, we conducted another 500 structural searches, using the *D*_3*h*_ cage as the seed structure. No structures with lower energies were found. All the local minima were verified via harmonic vibrational frequency calculations. The frozen-core approximation was employed for the inner shells of [1*s*^2^] for B and [1*s*^2^−4*d*^10^] for the La atoms. The zero-order regular approximation^49^ was applied, to account for the scalar relativistic effects. Low-lying isomers were subsequently optimized using the PBE and PBE0 density functionals^50^ along with the TZP basis sets. Born–Oppenheimer molecular dynamic simulations were further carried out on La_3_B_18_^–^ for 13 ps using the CP2K code^51^ at different temperatures, from 300 to 1000 K (Supplementary Fig. 4). To minimize the 4*f*-electron induced complexity (i.e. spin multiplicity) and considering the negligible geometry change due to the occupations of the localized 4*f* orbitals (radial-density maximum probability radii <0.5 Å), we used the 4*f*-in-core pseudopotentials^34^ for the lanthanide elements to optimize the geometric parameters in the other Ln_3_B_18_^–^ (Ln = Ce–Lu) species.

The simulation of the PE spectra was done using the ΔSCF-TDDFT method^52^ with the SAOP exchange-correlation functional^53^ to account for the long-range interactions. The ground state adiabatic and vertical detachment energies were calculated at the DFT levels, as well as the more accurate DLPNO-CCSD(T) level^54^ with the Def2-TZVP basis sets^55^ and the Def2-TZVPP pseudopotential for La^34^, utilizing the AutoAux generation procedure^55^. We also used the 4*f*-in-core pseudopotential^34^ for the simulation of the PE spectrum of Tb_3_B_18_^–^ without consideration of the 4*f* electron detachment channels. Previous studies showed that such detachment channels carried very low detachment cross sections at the low detachment photon energies used and the main PES features of Ln–B binary clusters were dominated by MOs with Ln *sd* or B *sp* characters^31–33^. Chemical bonding and electronic structure analyses were carried out by canonical molecular orbital (MO) theory and the semi-localized AdNDP method^39^. We also performed calculations using the energy decomposition analysis–natural orbitals for chemical valence (EDA–NOCV) approach^38^ to quantitatively elucidate the bonding mechanisms between the B_18_ and La_3_^–^ moieties. The bond order indexes of different interatomic interactions were calculated using the Mayer^56^, Gopinathan–Jug (G–J)^57^, and Nalewajski–Mrozek schemes^58^.

## Supplementary information

Supplementary Information

Description of Additional Supplementary Files

Supplementary Data 1

## Data Availability

The data that support the findings of this study are available within the article and the associated Supplementary information. Any other data are available from the corresponding authors upon request.

## Code availability

The TGMin code used for the global minimum search is available from the corresponding author (J.L.) upon request and signing a license.
