# Supplementary material for: Spherical trihedral metallo-borospherenes
Source: Nat Commun. 2020 Jun 2;11:2766. doi: 10.1038/s41467-020-16532-x (PMC7265489; doi:10.1038/s41467-020-16532-x)
Supplement: Supplementary file 1 — Supplementary Information [file 41467_2020_16532_MOESM1_ESM.pdf]

Supplementary Information

Spherical Trihedral Metallo-Borospherenes

Chen, et al.

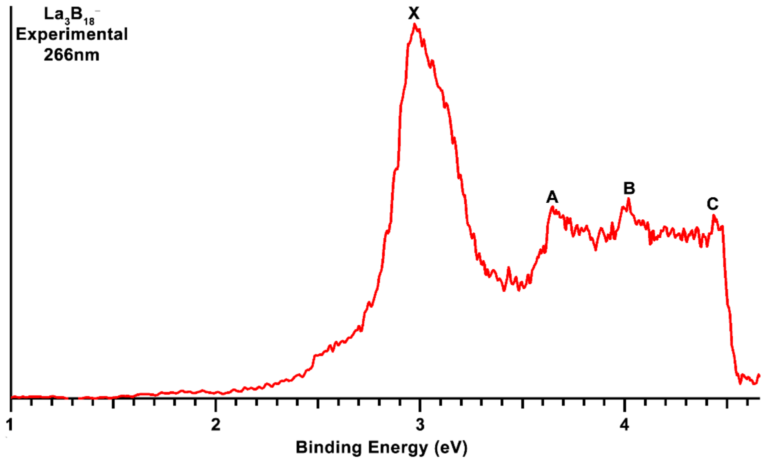

Supplementary Fig. 1. Photoelectron Spectrum of $La_3B_{18}^-$ at 266 nm.

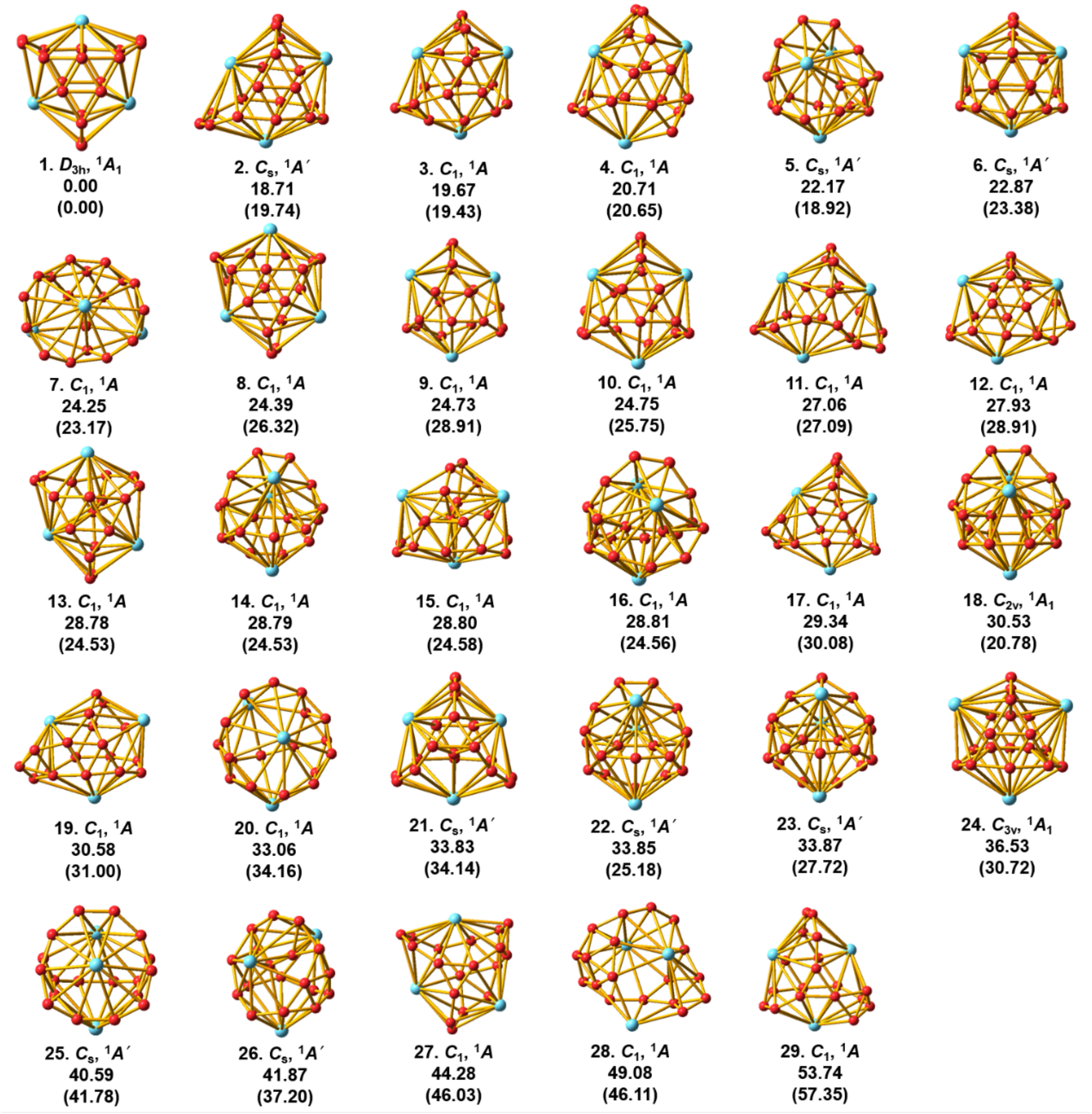

Supplementary Fig. 2. Low-lying isomers of $La_3B_{18}^-$ within 58 kcal mol^{-1} at the levels of PBE/TZP and PBE0/TZP (with parentheses).

a

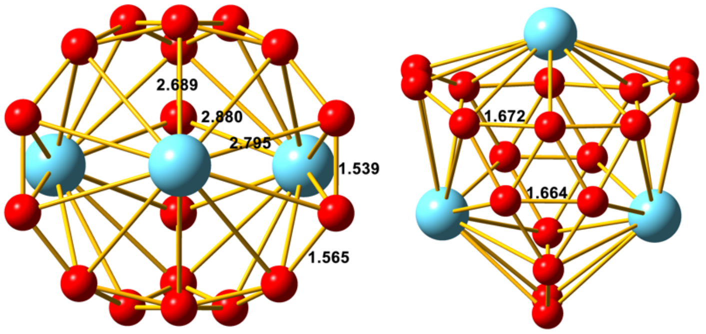

$La_3B_{18}^-$ (D_{3h}, $^1A_1$)

b

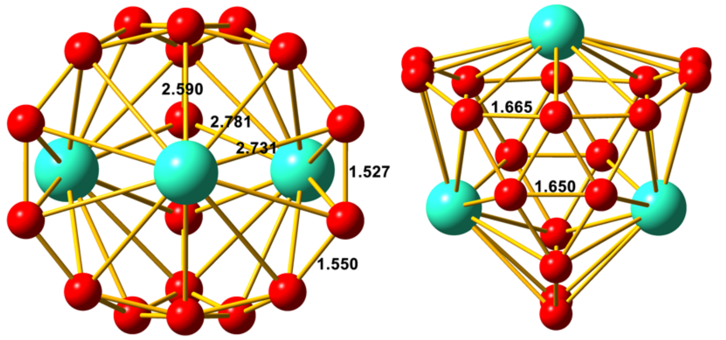

$Tb_3B_{18}^-$ (D_{3h})

Supplementary Fig. 3. The bond lengths in Å of the metallo-borophenes. **a** $La_3B_{18}^-$. **b** $Tb_3B_{18}^-$.

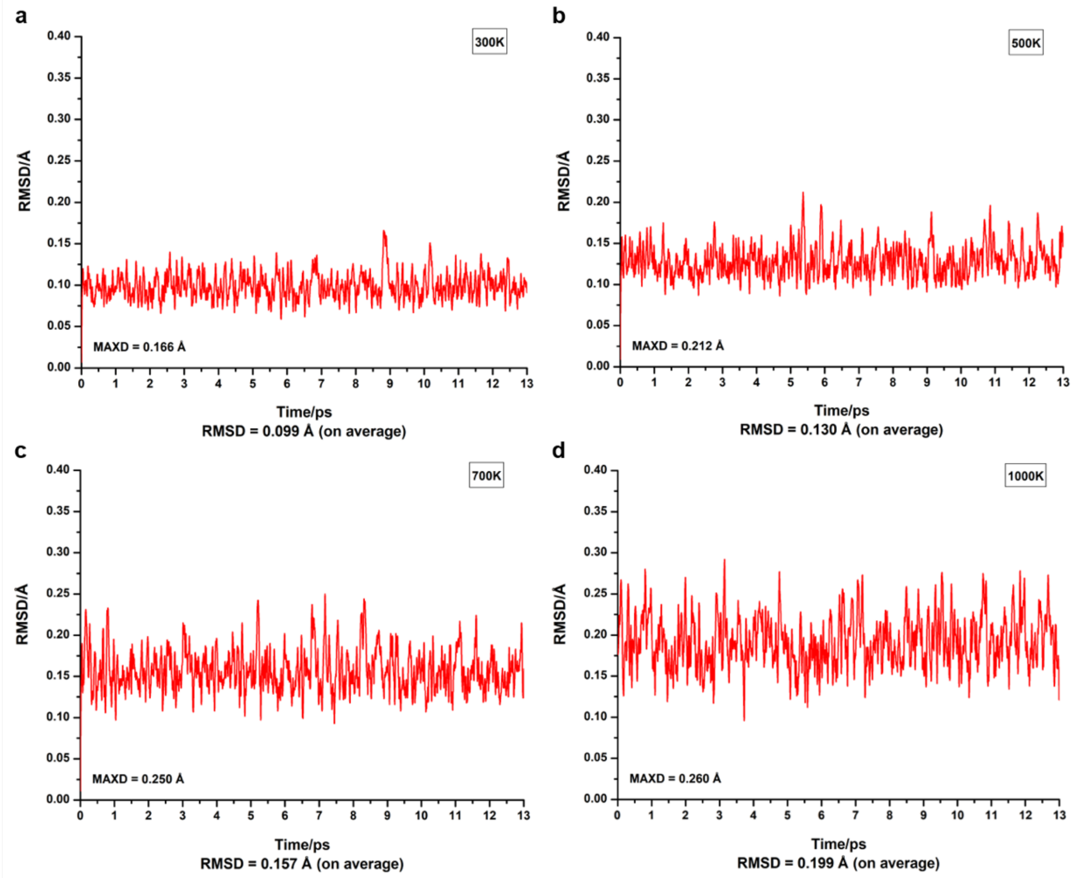

Supplementary Fig. 4. Born-Oppenheimer molecular dynamics simulations of the $La_3B_{18}^-$ cage. **a** At 300 K. **b** At 500 K. **c** At 700 K. **d** At 1,000 K. The root-mean-square-deviation (RMSD) and maximum bond length deviation (MAXD) values (on average) are indicated in Å.

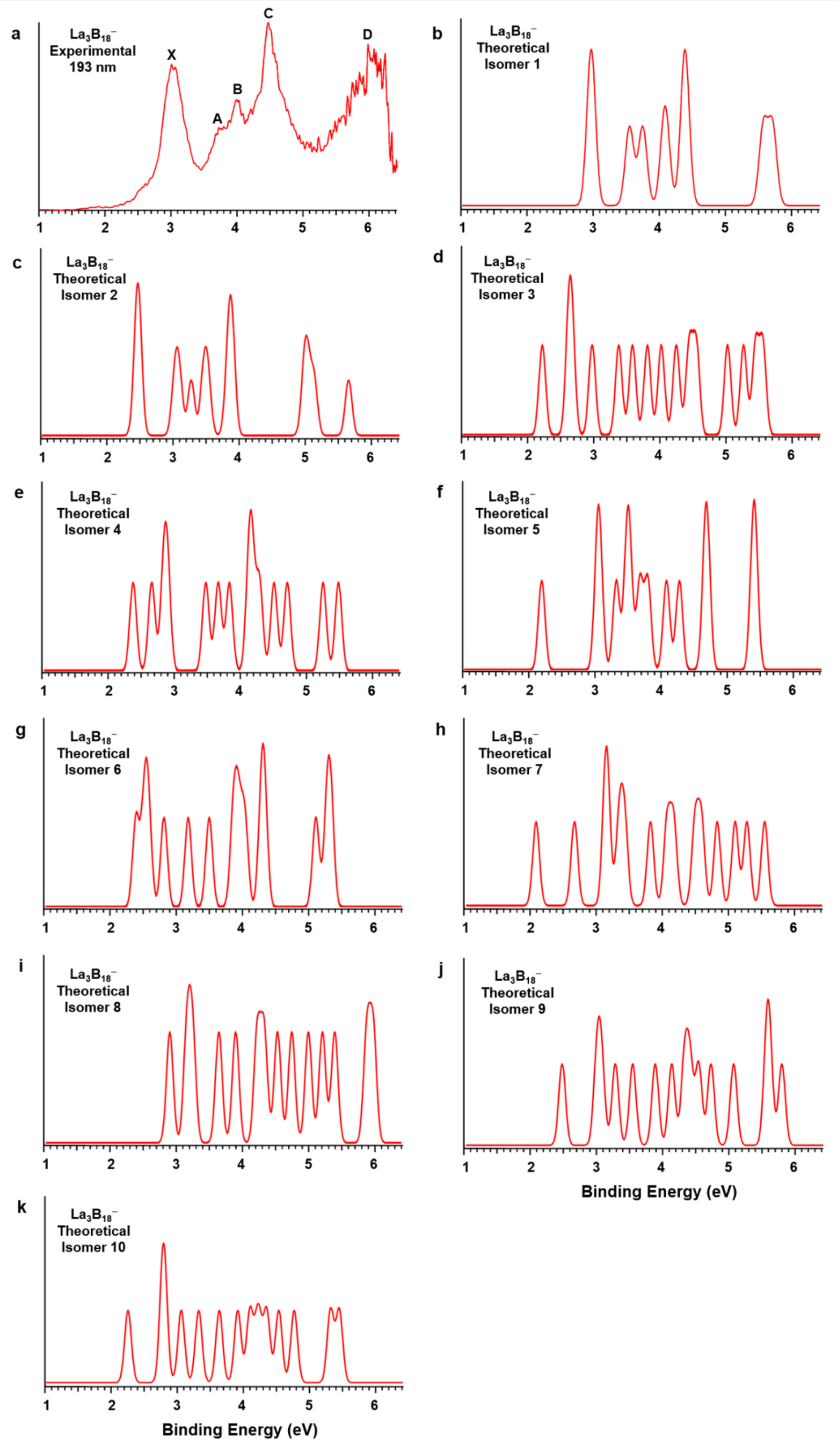

Supplementary Fig. 5. Comparison of experimental data with the theoretical calculations. **a** Photoelectron spectrum of $La_3B_{18}^-$ at 193 nm. **b-k** The simulated spectra of the first ten isomers of $La_3B_{18}^-$.

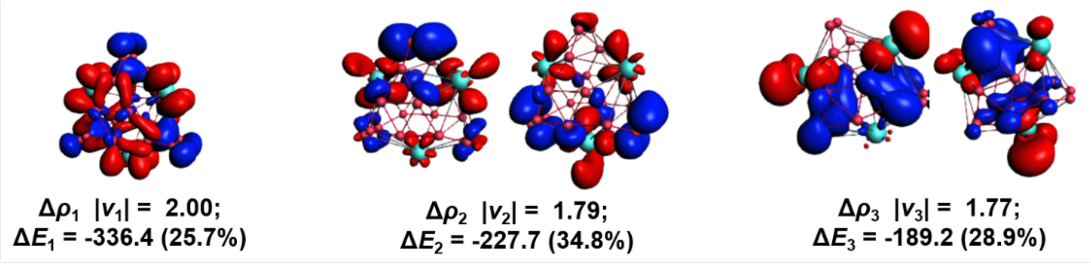

Supplementary Fig. 6. Plot of deformation densities $\Delta\rho_n$ (n = 1–3) (isovalue = 0.0025 a.u.) of the pairwise orbital interactions in $La_3B_{18}^-$ (D_{3h}, $^1A_1$), together with the associated interaction energies ΔE_n (in kcal mol^{-1}) and charge eigenvalues $|v_n|$ (in e). The charge flow direction is from red to blue.

Supplementary Table 1. Theoretical VDEs using ΔSCF-TDDFT for D_{3h} $La_3B_{18}^-$ in comparison with the experiment. The bold-face indicates the orbitals from which an electron is detached. All energies are in eV.

Feature	Expt. VDE	Electronic Configuration	Theo. VDE	Final State
X[a]	2.97(1)	$\ldots 6e'^4 7e'^4 4e''^4 8e'^4 5a_2''^2 7a_1'^2 1a_1''^2 2a_2'^2 9e'^4 8a_1'^2 \mathbf{5e''^3}$	2.972	$^2E''$
		$\ldots 6e'^4 7e'^4 4e''^4 8e'^4 5a_2''^2 7a_1'^2 1a_1''^2 2a_2'^2 9e'^4 \mathbf{8a_1'^1} 5e''^4$	2.987	$^2A_1''$
A	3.64(8)	$\ldots 6e'^4 7e'^4 4e''^4 8e'^4 5a_2''^2 7a_1'^2 1a_1''^2 2a_2'^2 \mathbf{9e'^3} 8a_1'^2 5e''^4$	3.551	$^2E''$
		$\ldots 6e'^4 7e'^4 4e''^4 8e'^4 5a_2''^2 7a_1'^2 1a_1''^2 \mathbf{2a_2'^1} 9e'^4 8a_1'^2 5e''^4$	3.747	$^2A_2''$
B	4.01(8)	$\ldots 6e'^4 7e'^4 4e''^4 8e'^4 5a_2''^2 7a_1'^2 \mathbf{1a_1''^1} 2a_2'^2 9e'^4 8a_1'^2 5e''^4$	4.066	$^2A_1''$
		$\ldots 6e'^4 7e'^4 4e''^4 8e'^4 5a_2''^2 \mathbf{7a_1'^1} 1a_1''^2 2a_2'^2 9e'^4 8a_1'^2 5e''^4$	4.096	$^2A_1'$
C	4.43(8)	$\ldots 6e'^4 7e'^4 4e''^4 \mathbf{8e'^3} 5a_2''^2 7a_1'^2 1a_1''^2 2a_2'^2 9e'^4 8a_1'^2 5e''^4$	4.368	$^2E'$
		$\ldots 6e'^4 7e'^4 4e''^4 8e'^4 \mathbf{5a_2''^1} 7a_1'^2 1a_1''^2 2a_2'^2 9e'^4 8a_1'^2 5e''^4$	4.386	$^2A_2''$
D	~6.0	$\ldots 6e'^4 7e'^4 \mathbf{4e''^3} 8e'^4 5a_2''^2 7a_1'^2 1a_1''^2 2a_2'^2 9e'^4 8a_1'^2 5e''^4$	5.547	$^2E''$
		$\ldots 6e'^4 \mathbf{7e'^3} 4e''^3 8e'^4 5a_2''^2 7a_1'^2 1a_1''^2 2a_2'^2 9e'^4 8a_1'^2 5e''^4$	5.684	$^2E'$

[a] The measured ADE for the X band or the EA of La_3B_{18} is 2.80 ± 0.05 eV.

Supplementary Table 2. Theoretical VDEs using ΔSCF-TDDFT for D_{3h} $Tb_3B_{18}^-$ in comparison with the experiment. The bold-face indicates the orbitals from which an electron is detached. Electron detachment from 4*f* orbitals, as well as the 4*f* spins, are not explicitly depicted due to the 4*f*-in-core pseudopotential. All energies are in eV.

Feature	Expt. VDE	Electronic Configuration	Theo. VDE	Final State[b]
X	3.26(3)	$\ldots 10e'^4 7e''^4 11e'^4 8a_2''^2 10a_1'^2 2a_2'^2 1a_1''^2 12e'^4 11a_1'^2 \mathbf{8e''^3}$	3.017	$^2E''$
		$\ldots 10e'^4 7e''^4 11e'^4 8a_2''^2 10a_1'^2 2a_2'^2 1a_1''^2 12e'^4 \mathbf{11a_1'^1} 8e''^4$	3.209	$^2A_1'$
A	3.84(3)	$\ldots 10e'^4 7e''^4 11e'^4 8a_2''^2 10a_1'^2 2a_2'^2 1a_1''^2 \mathbf{12e'^3} 11a_1'^2 8e''^4$	3.803	$^2E'$
B	4.28(6)	$\ldots 10e'^4 7e''^4 11e'^4 8a_2''^2 10a_1'^2 2a_2'^2 \mathbf{1a_1''^1} 12e'^4 11a_1'^2 8e''^4$	4.232	$^2A_1''$
		$\ldots 10e'^4 7e''^4 11e'^4 8a_2''^2 10a_1'^2 \mathbf{2a_2'^1} 1a_1''^2 12e'^4 11a_1'^2 8e''^4$	4.481	$^2A_2'$
C	4.52(7)	$\ldots 10e'^4 7e''^4 11e'^4 \mathbf{8a_2''^1} 10a_1'^2 2a_2'^2 1a_1''^2 12e'^4 11a_1'^2 8e''^4$	4.682	$^2A_2''$
		$\ldots 10e'^4 7e''^4 11e'^4 8a_2''^2 \mathbf{10a_1'^1} 2a_2'^2 1a_1''^2 12e'^4 11a_1'^2 8e''^4$	4.693	$^2A_1'$
D	4.77(5)	$\ldots 10e'^4 7e''^4 \mathbf{11e'^3} 8a_2''^2 10a_1'^2 2a_2'^2 1a_1''^2 12e'^4 11a_1'^2 8e''^4$	4.832	$^2E'$
E	~6.20	$\ldots 10e'^4 \mathbf{7e''^3} 11e'^4 8a_2''^2 10a_1'^2 2a_2'^2 1a_1''^2 12e'^4 11a_1'^2 8e''^4$	6.255	$^2E''$

[a] The measured ADE for the X band or the EA of Tb_3B_{18} is 3.13 ± 0.05 eV.

[b]The spin states are not the true final state spin-states because the 4f spins are not counted.

Supplementary Table 3. Relative energies (in kcal/mol) of the ferromagnetic (FM) and antiferromagnetic (AFM) $Pr_3B_{18}^-$ and $Tb_3B_{18}^-$ clusters at the PBE/TZP level using the broken-symmetry method.[a]

	$Pr_3B_{18}^-$			$Tb_3B_{18}^-$		
	2S + 1	Configuration	ΔE	2S + 1	Configuration	ΔE
FM	7	↑↑ ↑↑ ↑↑	0.00	19	↑↑↑↑↑↑ ↑↑↑↑↑↑ ↑↑↑↑↑↑	0.00
AFM	1	↑↑ ↓↓ ↑↓	8.88	7	↑↑↑↑↑↑ ↑↑↑↑↑↑ ↓↓↓↓↓↓	0.26
	3	↑↑ ↑↑ ↓↓	0.11	13	↑↓↑↑↑↑ ↑↓↑↑↑↑ ↑↓↑↑↑↑	14.24
	3	↑↑ ↑↓ ↑↓	19.34	15	↑↑↑↑↑↑ ↑↓↑↑↑↑ ↑↓↑↑↑↑	8.38
	5	↑↑ ↑↑ ↑↓	9.19	17	↑↑↑↑↑↑ ↑↑↑↑↑↑ ↑↓↑↑↑↑	12.47

[a] The up- and down-arrow indicate the majority and minority spins, respectively.

Supplementary Table 4. The ADE and VDE_1 values (eV) calculated using the PBE/TZP, PBE0/TZP, and DLPNO-CCSD(T)/Def2-TZVP methods, compared with the experimental data.

	$La_3B_{18}^-$				$Tb_3B_{18}^-$ [a]			
	PBE	PBE0	CCSD(T)	Expt.	PBE	PBE0	CCSD(T)	Expt.
ADE	2.800	2.753	2.828	2.80	2.832	2.816	2.901	3.13
VDE_1	2.983	2.858	2.972	2.97	3.058	3.003	3.017	3.26

[a] Computed using the 4*f*-in-core pseudopotential.

Supplementary Table 5. The major contributions from the La_3^- and B_{18} fragments to the 5*e*'', 9*e*' and 1a_1'' MOs of $La_3B_{18}^-$ with La–B bonding.

	Contribution
5e''	77% (B_{18} 4*e*'') + 10% (La_3^- 6*e*'') + 4% (La_3^- 4*e*'')
9e'	62% (B_{18} 6*e*') + 18% (La_3^- 12*e*') + 7% (La_3^- 10*e*')
1a_1''	71% (B_{18} 1a_1'') + 28% (La_3^- 2a_1'')

Supplementary Table 6. The NICS values (ppm) obtained from PBE0/VTZ method at different points of $La_3B_{18}^-$ (D_{3h}, $^1A_1$), compared with those of C_6H_6 and $[Zn^I]_8$.

		NICS (0)	NICS (1)
cage	$La_3B_{18}^-$ cage center	-47.87	-
	$[Zn^I]_8$ cubic center	-48.26	-
plane	$La_3B_{18}^-$ B_6 triangle	-31.44	-2.16
	C_6H_6	-8.52	-10.83
	$[Zn^I]_8$ face	-38.70	-9.71

Supplementary Table 7. Distances and bond order indices of different B-B and La-B interactions at PBE0/TZP level.

	Distance (Å)	Mayer	G-J	N-M(1)	N-M(3)
B-B (B_6 triangle)	1.683	0.58	0.66	0.69	0.70
	1.662	0.86	0.81	0.85	0.88
B-B (B_2 bridge)	1.572	1.36	1.32	1.35	1.44
	1.546	1.40	1.46	1.46	1.57
La-B	2.699	0.31	0.33	0.40	0.34
	2.807	0.30	0.27	0.32	0.29
	2.893	0.23	0.24	0.29	0.26

Supplementary Table 8. Cartesian coordinates of $La_3B_{18}^-$ (D_{3h}, $^1A_1$) and La_3B_{18} (C_s, $^1A'$) species at PBE0/TZP level.

$La_3B_{18}^-$ (D_{3h}, $^1A_1$)

1. La	2.337200	0.000000	0.000000
2. La	-1.168600	2.024074	0.000000
3. La	-1.168600	-2.024074	0.000000
4. B	1.468003	2.542656	-0.769276
5. B	-1.877762	0.000000	1.921668
6. B	1.468003	2.542656	0.769276
7. B	-0.480210	-0.831749	-2.309663
8. B	0.938881	-1.626190	-1.921668
9. B	0.938881	-1.626190	1.921668
10. B	1.468003	-2.542656	-0.769276
11. B	0.960421	0.000000	-2.309663
12. B	0.938881	1.626190	-1.921668
13. B	0.960421	0.000000	2.309663
14. B	-0.480210	-0.831749	2.309663
15. B	0.938881	1.626190	1.921668
16. B	-0.480210	0.831749	2.309663
17. B	-1.877762	0.000000	-1.921668
18. B	-0.480210	0.831749	-2.309663
19. B	-2.936006	0.000000	-0.769276
20. B	-2.936006	0.000000	0.769276
21. B	1.468003	-2.542656	0.769276

La_3B_{18} (C_s, $^2A'$)

1. La	-0.016425	-2.365100	0.000000
2. La	0.008284	1.180992	2.106492
3. La	0.008284	1.180992	-2.106492
4. B	0.758617	-1.478481	2.562359
5. B	-1.904206	1.894803	0.000000
6. B	-0.778848	-1.467601	2.562150
7. B	2.307861	0.451204	-0.836764
8. B	1.913546	-0.942641	-1.647120
9. B	-1.926531	-0.915779	-1.647186
10. B	0.758617	-1.478481	-2.562359
11. B	2.311098	-0.968022	0.000000
12. B	1.913546	-0.942641	1.647120
13. B	-2.324228	-0.935631	0.000000
14. B	-2.301391	0.483309	-0.836807
15. B	-1.926531	-0.915779	1.647186
16. B	-2.301391	0.483309	0.836807
17. B	1.930295	1.867948	0.000000
18. B	2.307861	0.451204	0.836764
19. B	0.783978	2.940625	0.000000
20. B	-0.743071	2.951326	0.000000
21. B	-0.778848	-1.467601	-2.562150
