# Supplementary material for: Spherical trihedral metallo-borospherenes
Source: Nat Commun. 2020 Jun 2;11:2766. doi: 10.1038/s41467-020-16532-x (PMC7265489; doi:10.1038/s41467-020-16532-x)
Supplement: Supplementary file 2 — Description of Additional Supplementary Files [file 41467_2020_16532_MOESM2_ESM.pdf]

Description of Additional Supplementary Files

Supplementary Data 1. Cartesian coordinates of D3h Ln3B18Đ (Ce Đ Lu) with 4f-in-core pseudopotential and the corresponding basis sets at the PBE0 level.
